# Decreased Expression of KIFC1 in Human Testes with Globozoospermic Defects

**DOI:** 10.3390/genes7100075

**Published:** 2016-09-27

**Authors:** Erlei Zhi, Peng Li, Huixing Chen, Peng Xu, Xiaobin Zhu, Zijue Zhu, Zuping He, Zheng Li

**Affiliations:** 1 Department of Andrology, Center for Men’s Health, Urologic Medical Center, Shanghai General Hospital, Shanghai Jiao Tong University, 100 Haining Rd, Shanghai 200080, China; zhzzer1985@sjtu.edu.cn (E.Z.); fengzhongjiezou@163.com (P.L.); chenhuixing11111@163.com (H.C.); zhuzijue@126.com (Z.Z.); 2 Department of ART, Institute of Urology, Urologic Medical Center, Shanghai General Hospital, Shanghai Jiao Tong University, 100 Haining Rd, Shanghai 200080, China; 3 Oriental Medical Group Jinghua Hospital of Sheng yang, 156 Nanjing Rd, Shengyang 110000, China; xupeng0699@163.com; 4 IVF Unit, Department of Obstetrics and Gynecology, Ruijin Hospital Affiliated to Shanghai Jiao Tong University, 197 Ruijin Er Rd, Shanghai 200025, China; cliff26@126.com; 5 State Key Laboratory of Oncogenes and Related Genes, Renji-Med X Clinical Stem Cell Research Center, Ren Ji Hospital, School of Medicine, Shanghai Jiao Tong University,160 Pujian Road, Shanghai 200127, China

**Keywords:** KIFC1, globozoospermic defects, male infertility, kinesin14 family

## Abstract

Globozoospermia is a rare (prevalence of <0.1%) but severe male infertility condition. In our previous study, we found that robust KIFC1 immunostaining was detected in the human elongating/elongated spermatids during human acrosomogenesis. However, the relationship between the decreased expression of KIFC1 and human globozoospermia remains largely unknown. Testicular biopsies of 30 globozoospermia and 30 obstructive azoospermia patients who underwent infertility evaluation and treatment were utilized in this study. Reverse transcriptase polymerase chain reaction (RT-PCR), Western blots, immunohistochemistry, an in vivo model, and intratesticular injection of small inhibitory RNA (siRNA) against the *Kifc1* gene were employed, and sperm abnormalities were evaluated by hematoxylin and eosin (H&E) staining and immunocytochemistry. We revealed that the testicular level of *KIFC1* mRNA in globozoospermia was significantly reduced compared with that in obstructive azoospermia, and the KIFC1 protein was barely detectable in testicular specimens in 30% (9 of 30) of patients with globozoospermia. Furthermore, knockdown of the *Kifc1* gene in mice increased the percentage of sperm with globozoospermic defects (26.5%). Decreased KIFC1 expression was mainly observed in the testes of patients with globozoospermia at the spermatid stage, which may be useful for counseling and management of such patients.

## 1. Introduction

Globozoospermia is a rare (prevalence of <0.1%) but severe fertility disorder characterized by round-headed spermatozoa with malformed acrosomes or a complete lack of acrosomes [1]. According to the percentage of round-headed and acrosomeless spermatozoa per ejaculate, globozoospermia can be classified as classic/total globozoospermia (100%) or partial globozoospermia (<100%) [2]. Notably, the exact molecular mechanisms underlying this condition remain largely unknown.

Mutations in several genes are associated with the globozoospermic phenotype; these genes include *Mfsd14a* [3], *Atg7* [4], *Smap2* [5], *Spaca1* [6], *Dpy19l2* [7], *Hsp191* [8], *Vps54* [9], *Gopc* [10], *Pick1* [11], *Agfg1* [12], and *Csnk2a2* [13]. Similarly, causative mutations for globozoospermia have been identified in humans, including those in *SPATA16* [14], *DPY19L2* [15,16], and *PICK1* [10].

KIFC1, a member of the kinesin-14 family, was first identified in the mouse brain and embryos, but its levels are highest in adult testes [17]. KIFC1 is the human homolog of *kar3* in yeast, *ncd* in *Drosophila*, *xctk2* in *Xenopuslaevis*, and *Cho2* in rats. Previous studies have found that KIFC1, as a motor protein, participates in acrosomogenesis in invertebrates and mice. For example, KIFC1 is involved in acrosome formation in *Eriocheir sinensis* [18] and cell morphological changes in *Octopus tankahkeei* [19]. KIFC1 also drives acrosome formation and cell morphological changes by interacting with the AFS (Acroframosome) and LCx (Lamellar Complex) during acrosomogenesis in *Macrobrachium nipponense* [20]. Based on the colocalization of KIFC1 and importin β, KIFC1 has been found to be associated with the acrosome from the initial stages of development in mice [21]. In our previous study, we have found that the expression patterns of the *KIFC1* gene are changed during human spermiogenesis and that this gene is highly expressed at the spermatid stage [22]. Therefore, we hypothesized that KIFC1 might play an important role in human acrosomogenesis, and that decreased expression of KIFC1 in human testes would lead to globozoospermic defects.

In order to investigate the function of KIFC1 in human acrosomogenesis, we examined specimens obtained from testicular biopsies of patients with globozoospermia and obstructive azoospermia, and compared the expression of KIFC1 in the testes of these patients. We also knocked down the *Kifc1* gene in testes of 3-week-old mice to determine the role of KIFC1 in regulating acrosomogenesis.

## 2. Materials and Methods

### 2.1. Patients and Samples

Patients with globozoospermia and obstructive azoospermia (n = 30 and 30, respectively) were recruited between February 2013 and December 2015, and testicular tissue specimens were obtained by biopsy. Exclusion criteria included abnormal karyotype, Y chromosome microdeletion, hormone treatment at the time of biopsy, exposure to alcohol, drugs, or surgery during the previous 3 months, presence of systemic diseases such as diabetes or hypertension, and a history of vasectomy.

Prior to biopsy, demographic information was obtained for each patient. Testis sizes were measured by ultrasound examination, and semen was analyzed. Serum levels of follicle-stimulating hormone (FSH), leuteinizing hormone (LH), testosterone (T), prolactin (PRL), and estradiol (E2) were measured by chemiluminescence assay.

### 2.2. RNA Extraction and Reverse Transcriptase Polymerase Chain Reaction (RT-PCR)

RNA was extracted using the RNeasy Micro kit (Qiagen, Valencia, CA, USA) according to the manufacturer’s instruction. The precipitated RNA was dissolved in 14 µl of RNase-free water, and the RNA concentration was measured at 260 nm in a spectrophotometer, whereas purity was assessed using the A_260_/A_280_ ratio. Samples were stored at −80 °C until use. Reverse transcription was carried out using a kit (Thermo Scientific, Dalian, China) under the following conditions: 42 °C for 60 min, followed by 70 °C for 5 min. The product was stored at −20 °C for PCR, which was performed under the following conditions: 94 °C for 5 min; 28 cycles of 94 °C for 30 s, 55 °C for 30 s, and 72 °C for 30 s; and 72 °C for 10 min. Human *ACTIN* was used as an internal control.

### 2.3. SDS-PAGE and Immunoblot Analysis

Testicular tissue was homogenized in radio-immunoprecipitation assay lysis buffer (Solarbio, Shanghai, China) containing protease inhibitors. The lysate was centrifuged at 12,000 rpm for 20 min at 4 °C. After removal of the supernatant, 1× loading buffer was added to the sample. Protein concentration was measured using a bicinchoninic acid protein assay kit (Qiagen) according to the manufacturer’s instructions. Approximately 30 μg of protein was loaded on each gel and electrotransferred to polyvinylidene difluoride membranes using standard procedures. KIFC1 was detected using a rabbit polyclonal antibody to KIFC1 (1:200; Proteintech, Chicago, IL, USA), followed by a mouse anti-rabbit secondary antibody (1:3000; MultiSciencesBiotech, Chicago, IL, USA). Band intensity was quantified relative to that of β-actin (MultiSciencesBiotech) using Image Quant TL 7.0 software (GE Healthcare, Little Chalfont, UK).

### 2.4. Immunohistochemistry and Immunocytochemistry

Immunohistochemical detection of the KIFC1 protein was carried out as previously described [23]. Testicular tissue was fixed in 4% paraformaldehyde overnight, washed three times for 15 min with phosphate-buffered saline (PBS), and incubated overnight in 0.5 M sucrose in PBS. Tissue samples were embedded in Tissue-Tek O.C.T. Compound (Sakura Finetek, Torrance, CA, USA); tissue blocks were stored at −80 °C until use, and cut into 5 µm frozen sections using a cryostat. Tissue sections were incubated with anti-KIFC1 (1:200) (Proteintech) in 5% BSA blocking buffer at 4 °C overnight. After washing three times in PBS containing 0.1% Triton X-100 for 45 min, samples were incubated with fluorescein isothiocyanate-conjugated anti-mouse IgG (1:200; Invitrogen, Carlsbad, CA ,USA) for 1 h, and the epididymal sperm were incubated with peanut agglutinin (PNA) (1:300; Invitrogen) for 30 min at room temperature. The samples were washed three times in PBS and incubated with DAPI (1:200; Invitrogen), mounted with Vectashield (Vector Laboratories, Burlingame, CA, USA) and imaged by fluorescence microscopy (Carl Zeiss, Jena, Germany). Immunocytochemistry was performed with a method similar to the immunohistochemistry except that the starting samples were cells.

### 2.5. Kifc1 Gene Knockdown and Evaluation of Epididymal Sperm Morphology

Three double-stranded Stealth short interfering (si)RNAs (Biotend, Shanghai, China) against *Kifc1* were diluted to 20 μM with RNase-free water. The knockdown efficiency of the siRNAs was evaluated by Western blotting and immunocytochemistry after 72 h. The sequence with the highest knockdown efficiency (siRNA1: 5′-CGAGUUACGUAGAGAUCUAdTdT-3′) was selected for in vivo studies.

Intratesticular injection of siRNA was performed as previously described [24]. Briefly, 3-week-old male mice were anesthetized, and their testes were exteriorized through a 3-cm midline abdominal incision. Approximately 4 µl of siRNA1 mixed with indicator dye (0.3% trypan blue) were injected into the seminiferous tubules via the rete testis. An equal amount of control siRNA (Biotend, Shanghai, China) was injected into the other testis. The testes were replaced and the incision was sutured, and the efficiency of knockdown in vivo was evaluated by Western blotting 72 h after siRNA1 injection. Epididymal sperm morphology was analyzed 3 weeks later.

### 2.6. Hematoxylin and Eosin Staining

Spermatozoa were fixed in 4% formaldehyde for 24 h at 4 °C as previously described [25]. Deparaffinized and rehydrated specimens were stained with hematoxylin for 15 min, followed by eosin for 10 min at room temperature, and were then mounted using neutral resin and visualized by light microscopy (total of 20 visual fields imaged; at least 200 cells per visual field were counted for calculation of the acrosome defect ratio).

### 2.7. Statistical Analysis

Statistical analysis was performed using SPSS software (V19.0; IDM, Chicago, IL, USA). *p* values were calculated by two-sided Student’s *t*-test to evaluate the differences between groups. *p* < 0.05 was considered statistically significant, and *p* < 0.001 was extremely statistically different.

### 2.8. Ethics Statement

This study was approved by the Ethical Review Committee of Ren Ji Hospital (license number of ethics statement: 2012-01) and the institutional animal care and use committee of Shanghai Jiao Tong University School of Medicine. All participants provided written, informed consent (SYXK 2008-0050).

## 3. Results

### 3.1. Clinical Characteristics of Globozoospermia and Obstructive Azoospermia

The patients with obstructive azoospermia exhibited typical epididymal obstruction and changes in the mesh of the epididymis ducts, as determined by ultrasound (Figure S1), and the globozoospermia patients showed spermatozoa that were round-headed and that lacked an acrosome (>70%) (Figure S2). There was no obvious difference in age, testicular volume, or the levels of FSH, LH, T, and E2 between the globozoospermia and obstructive azoospermia patients (Table S1).

### 3.2. KIFC1 Expression in the Testes of Patients with Globozoospermia and Obstructive Azoospermia

**Figure 1:**
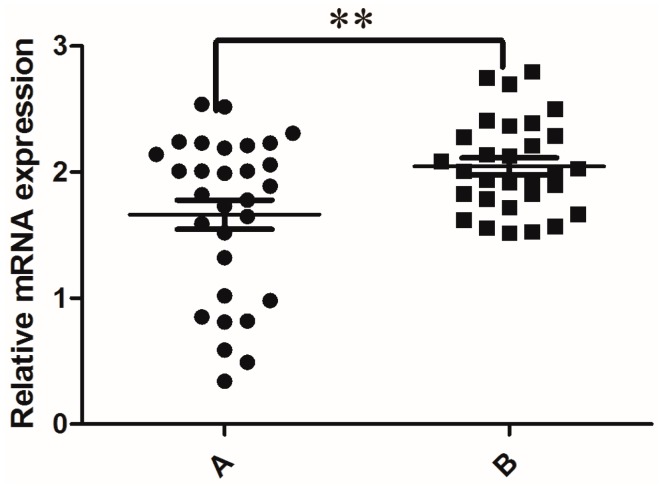
Relative human testicular mRNA expression of *KIFC1*. Gene expression was normalized to the expression of *ACTIN*. ** *p* < 0.001. This experiment was repeated three times. A: Globozoospermia; B: Obstructive azoospermia.

**Figure 2:**
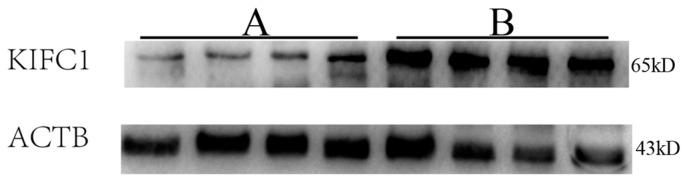
Representative gels showing KIFC1 protein levels in human testes with globozoospermia and obstructive azoospermia. ACTB was used as a loading control of total proteins. This experiment was repeated three times. A: Globozoospermia; B: Obstructive azoospermia.

**Figure 3:**
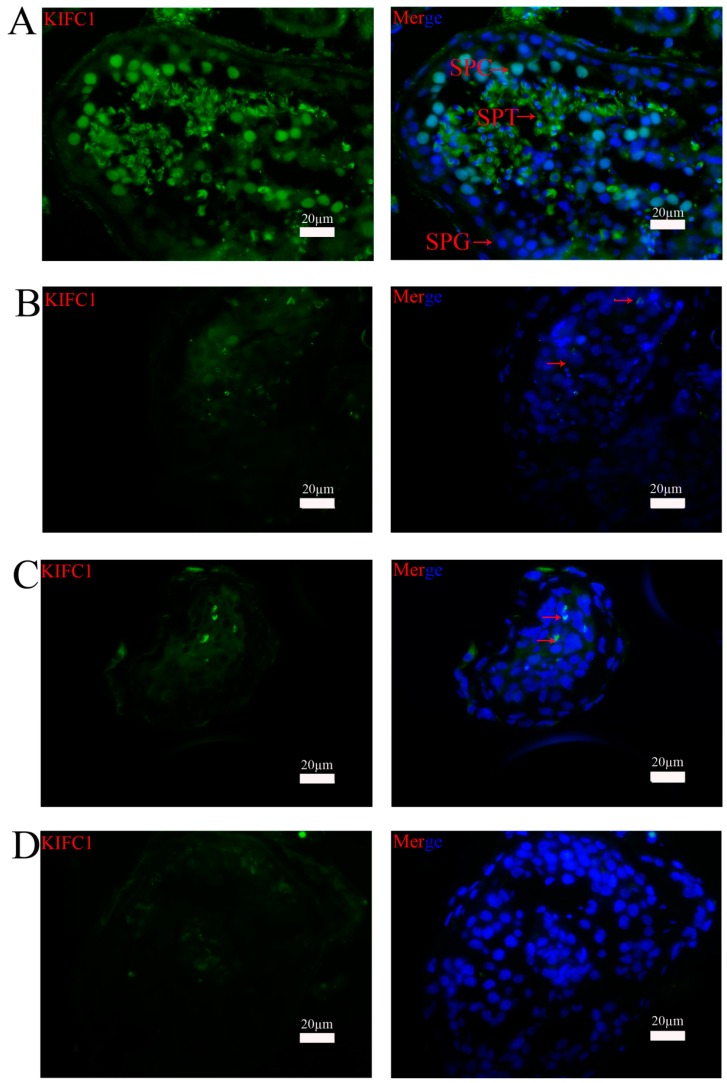
Immunohistochemical staining of the expression of KIFC1 protein in the testes of human patients with globozoospermia and obstructive azoospermia. (**A**) Testicular tissue sections of a representative patient with obstructive azoospermia. A high level of KIFC1 immunostaining was found in the round and elongating/elongated spermatids, with weaker expression in pachytene spermatocyte nuclei, and no expression in spermatogonia or Sertoli cells. (**B**), (**C**) Testicular tissue section of two representative patients with globozoospermia. KIFC1 protein was absent or barely detectable. (**D**) Negative control. KIFC1-positive cells are indicated by arrows. SPT: spermatids; SPC: spermatocytes; SPG: spermatogonia. Scale bars = 20 µm.

As shown in Figure 1, RT-PCR analysis revealed that nine of the thirty globozoospermia samples had significantly reduced expression of *KIFC1* mRNA compared with that in the obstructive azoospermia samples (*p* < 0.001). Western blots (Figure 2) and immunohistochemistry (Figure 3) were utilized to evaluate the expression levels of the KIFC1 protein and showed a concomitant decrease of KIFC1 protein in the testes of globozoospermia patients compared to obstructive azoospermia. Furthermore, we observed that KIFC1 was the most highly present in round and elongating/elongated spermatids, hardly expressed in pachytene spermatocytes, and undetected in spermatogonia and Sertoli cells of obstructive azoospermia. Robust KIFC1 immunnostaining was found in the round and elongating/elongated spermatids of obstructive azoospermia (Figure 3A). In contrast, the KIFC1 signal was absent or barely detectable in nine of thirty globozoospermia samples (Figure 3B,C). There were no significant differences between the other twenty-one globozoospermia samples and the obstructive azoospermia samples.

### 3.3. Kifc1 Knockdown Increased the Rate of Globozoospermic Sperm

**Figure 4:**
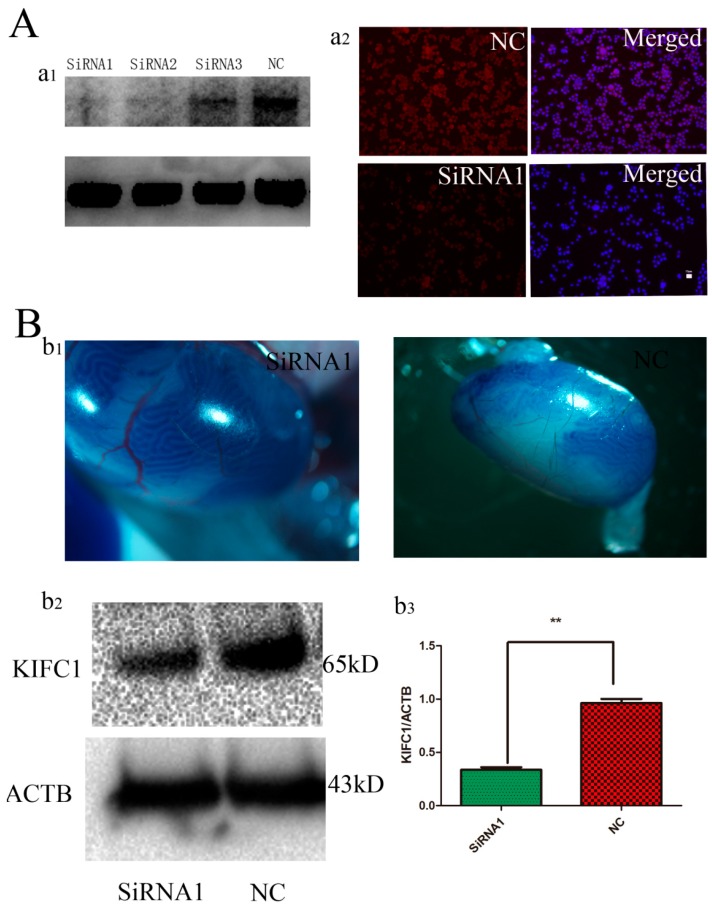
Identification of siRNAs targeting *Kifc1* effectively in vitro and in vivo. (**A, a_1_**) Western blot analysis showed the knockdown effect of *Kifc1* siRNAs on KIFC1 protein expression at 72 h. ACTB was used as a loading control. (**A, a_2_**) Immunocytochemistry analysis revealed the knockdown effect of *Kifc1* siRNA1 on KIFC1 protein expression in the GC-2 cell line. (**B, b_1_**) Trypan blue staining was used as a marker of siRNA injection; 85% of the seminiferous tubules were injected with siRNA. (**B, b_2_**) Western blot analysis of the KIFC1 protein expression in 3-week-old mouse testis 72 h after injection of *Kifc1* siRNA1. (**B, b_3_**) Quantification of Western blot analysis. More than 55% of KIFC1 was decreased after the injections. All the data were collected from three independent experiments.

**Figure 5:**
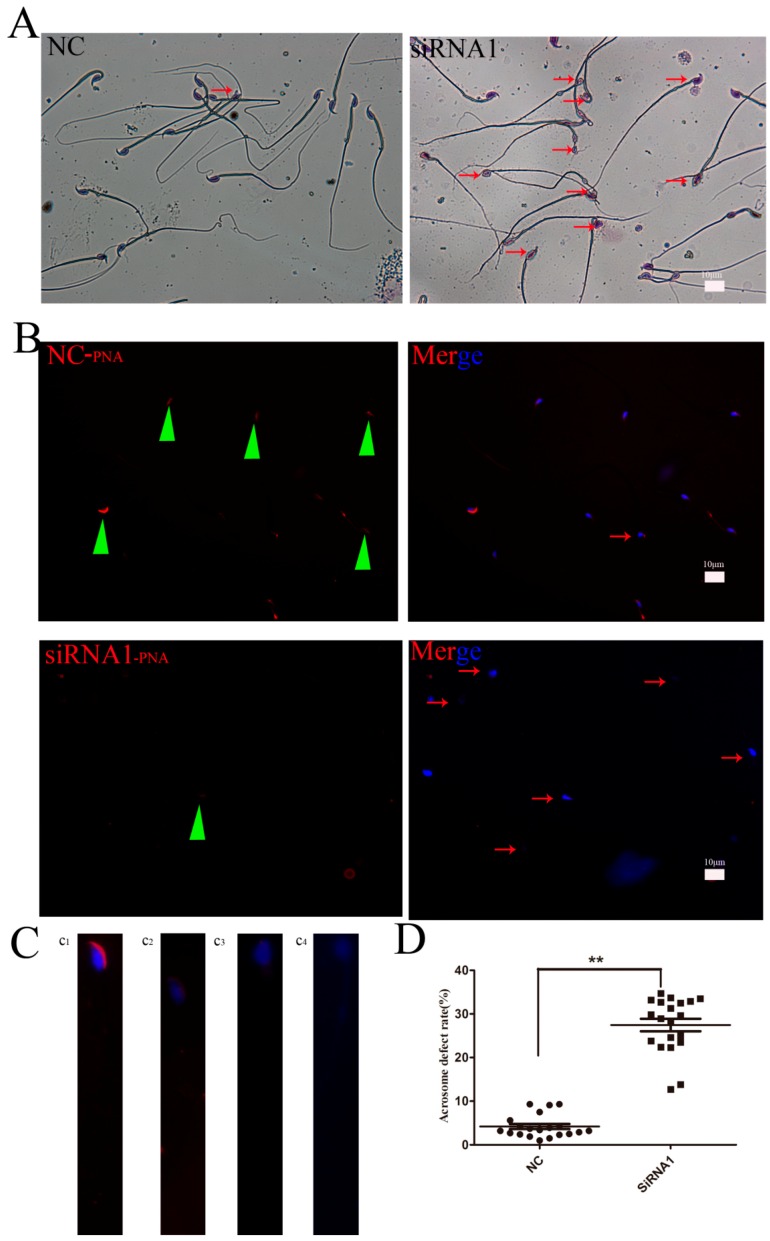
Knockdown of *Kifc1* in vivo affected the morphology of the sperm acrosome in mouse cauda epididymis. (**A**) H&E staining display that sperm treated with *Kifc1* siRNA1 had irregularly shaped round heads similar to those seen in human globozoospermia. (**B**)Representative images of PNA (green triangles) staining of abnormal sperm also demonstrate various round-headed and acrosomeless sperm (red arrows). (C, **c_1_**) The morphology of the normal acrosome (×100). (**C**, **c_2_, c_3_, c_4_**) The morphology of round-headed with acrosomeless sperm (×100). (**D**) The proportion of sperm with globozoospermic defects was markedly increased after treatment with *Kifc1* siRNA1 (*p* < 0.05). The results were representative of three independent experiments.

The efficiency of siRNAs against *KIFC1* transcripts was tested in GC-2 spd cells. At 72 h after transfection, *Kifc1* siRNA1 was found to be the most effective by Western blot analysis (Figure 4A, a_1_) and immunnostaining (Figure 4A, a_2_). Approximately 85% of the seminiferous tubules of the mice were internalized by the siRNA1, as observed by trypan blue staining (Figure 4B, b_1_), and the knockdown efficiency of KIFC1 was approximately 55% (Figure 4B, b_2_, b_3_). The morphology of sperm in the cauda epididymis was examined 3 weeks after intratesticular injection of *Kifc1* siRNA1. Compared with the control group, H&E staining showed that sperm treated with the *Kifc1* siRNA1 had irregularly shaped round heads similar to those seen in human globozoospermia (Figure 5A). PNA staining of abnormal sperm also demonstrated various round-headed and acrosomeless sperm, in addition to mislocalization and deformation (Figure 5C, c_2_, c_3_, c_4_), although normal sperm were also observed (Figure 5C, c_1_). The proportion of sperm with globozoospermic defects increased markedly to 26.5% upon treatment with *Kifc1* siRNA, and this percentage was significantly higher than that of the control (4%, *p* < 0.05, Figure 5D).

## 4. Discussion

The pathogenesis of globozoospermia most likely originates in spermiogenesis, specifically in acrosome formation and sperm head elongation. Acrosomogenesis is characterized by the dynamic flow of proteins and proacrosomal vesicles along the “ER-Golgi-Acrosome” pathway [26]. Numerous proacrosomic granules and unique proteins, such as acrosin and acrosin-binding protein (ACRB/OY-TES-1), are transported to and accumulate in the concave region of the nuclear surface [27,28]. Therefore, active trafficking from the Golgi apparatus is involved in acrosome formation. KIFC1, a minus-end motor protein, first associates with vesicles localized between the Golgi and the spermatid nucleus, followed by association with the growing acrosome [21], delivery of vesicles from the Golgi to the growing acrosome, continued transport along microtubules of the manchette [29], recycling of vesicles back to the Golgi [30], and linkage of the manchette to the nuclear membrane [31]. Previous studies have shown that the association of KIFC1 with Hrb (also known as hRIP or RAB) plays an important role in acrosomogenesis, and that homozygous deletion of the Hrb gene from mice results in globozoospermia [32]. However, the pathophysiological role of KIFC1 in human testes with globozoospermic defects has remained unclear. Our results were consistent with previous findings that KIFC1 levels are highest in round and elongating/elongated spermatids of the human testis. These data indicate that KIFC1 plays a pivotal role in human acrosomogenesis.

In the present study, expression analysis of KIFC1 by RT-PCR, immunohistochemistry, and Western blotting showed that KIFC1 was absent or undetectable in the testicular tissues of 30% (9 of 30) of patients with globozoospermia. KIFC1 levels were highest in round and elongating/elongated spermatids and were weakly expressed in pachytene spermatocyte nuclei of the human testis. Globozoospermia is identical to obstructive azoospermia, except for acrosogenesis. Therefore, the differential testicular expression of the KIFC1 in globozoospermic patients may reflect a true pathogenic mechanism. In our study, KIFC1 immunostaining was absent or hardly detectable in the round and elongating/elongated spermatids of testes from nine patients with globozoospermia. The normal presence of KIFC1 expression in the other twenty-one samples from patients with globozoospermia may be explained by other genes or environmental factors [33]. In order to provide evidence that the deficient expression of KIFC1 may be a true pathogenic factor for patients with globozoospermia, we performed a functional analysis by knocking down *Kifc1* expression in vivo in mice.

In mice, round spermatids begin to appear at approximately 3 weeks of age; transcription and translation mostly cease during this process, and siRNAs introduced into the seminiferous tubule at this time point would degrade the targeting mRNA, which could not be replaced by newly transcribed mRNA [34]. Thus, the protein level of the targeting gene will be reduced for a relatively long period. We injected *Kifc1* siRNAs into the testes of 3-week-old mice as previously described [35]. We used siRNA rather than dsRNA, which is easily phagocytosed and degraded by Sertoli cells [36]. H&E and PNA staining confirmed that sperm in *Kifc1* siRNA1-treated mice had irregularly shaped round heads with malformed acrosomes or no acrosomes at all, which was similar to human globozoospermia, and that the proportion of sperm with globozoospermic defects was increased markedly to 26.5%. This functional result indicates that KIFC1 plays an important role in acrosome biogenesis.

In conclusion, we have revealed a decrease in KIFC1 expression in human testes with globozoospermic defects. Knockdown of *Kifc1* in vivo resulted in round-headed spermatozoa with malformed acrosomes or without acrosomes. Therefore, we consider that deficient expression of KIFC1 can be a novel marker for the prediction of globozoospermic defects. Moreover, clarifying the biological function of KIFC1 may facilitate the development of approaches for patient counseling and management, especially in cases of globozoospermia.

## Supplementary Materials

The following are available online at www.mdpi.com/2073-4425/7/10/75/s1, Figure S1: Mesh change of epididymis ducts as determined by ultrasound, relevant mesh changes of epididymis duct are indicated by arrows, Figure S2: (A) Globozoospermia patients presented spermatozoa with round-heads that lacked an acrosome; (B) The normal spermatozoa with the intact acrosome, relevant round-headed spermatozoa are indicated by arrows, Table S1: Clinical characteristics of patients included in the study.
