# Supplementary material for: Decreased Expression of KIFC1 in Human Testes with Globozoospermic Defects
Source: Genes (Basel). 2016 Sep 27;7(10):75. doi: 10.3390/genes7100075 (PMC5083914; doi:10.3390/genes7100075)
Supplement: Supplementary file 1 [file genes-07-00075-s001.docx]

Supplementary materials: Decreased expression of KIFC1 in human testes with globozoospermic defects

Erlei Zhi, Peng Li, Huixing Chen, Xiaobin Zhu, Zijue Zhu, Peng Xu, Zuping He and Zheng Li


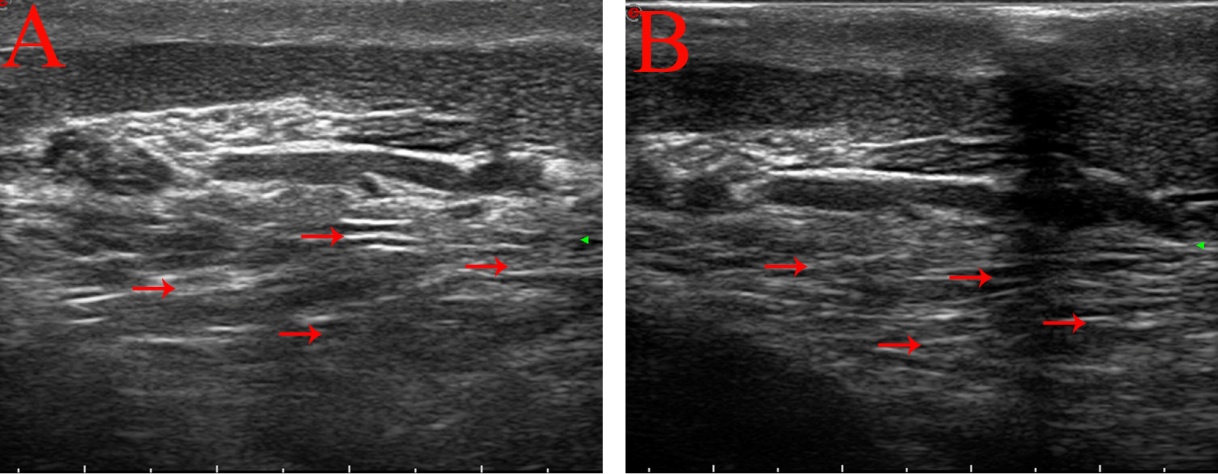


**Figure S1.** (**A, B**) Mesh change of epididymis ducts as determined by ultrasound. Relevantly mesh change of epididymis duct are indicated by arrows


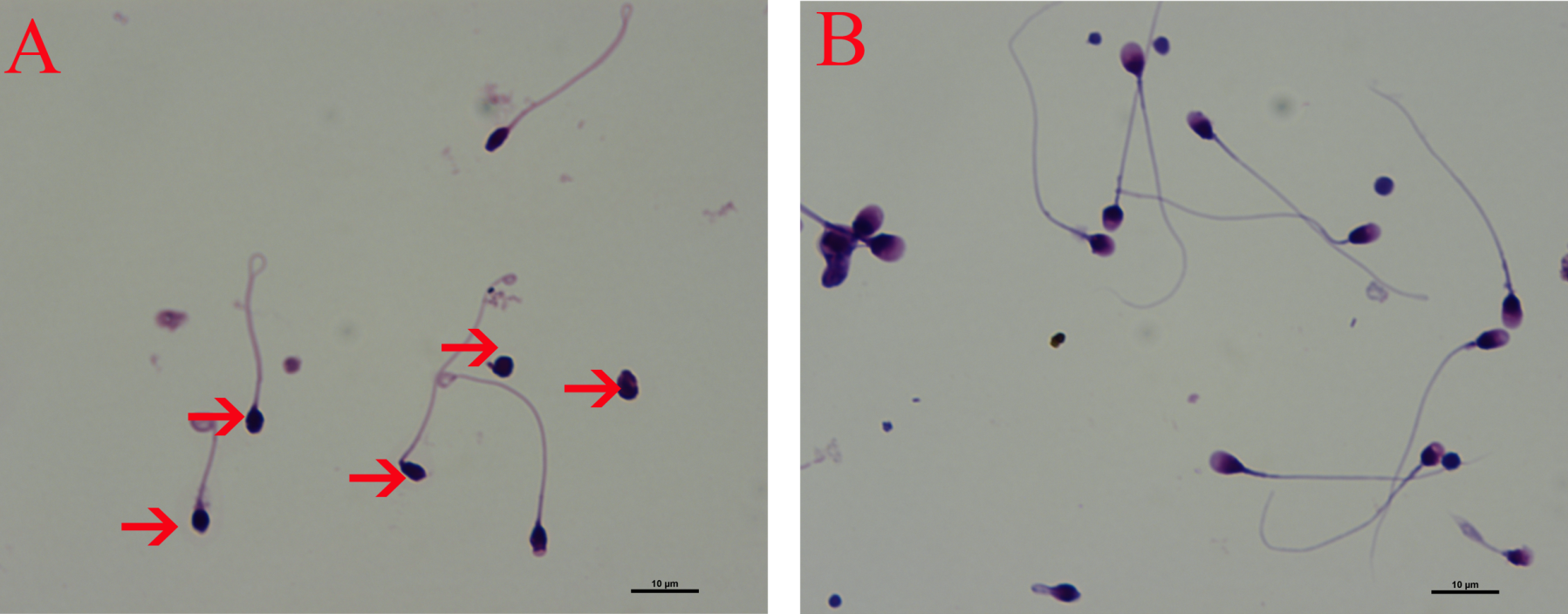


**Figure S2.** (**A**) Globozoospermia patients presented spermatozoa with round-headed spermatozoa that lacked an acrosome. (**B**) The normal spermatozoa with the intact acrosome. Relevantly round-headed spermatozoa are indicated by arrows

**Table S1.** Clinical characteristics of patients included in the study.

|  | A（n = 9） | B（n = 16） | *p* value |
| --- | --- | --- | --- |
| Age(y) | 29 ± 3.48 | 31 ± 5.25 | 0.07 |
| FSH(IU/L) | 5.2 ± 1.25 | 4.7 ± 3.82 | > 0.05 |
| LH(IU/L) | 5.0 ± 1.59 | 5.3 ± 2.15 | 0.075 |
| T(nmol/l) | 10 ± 3.15 | 9.5 ± 4.21 | 0.437 |
| E_2_(pmol/l) | 72 ± 0.19 | 70 ± 1.35 | 0.07 |
| Average testicular volume (mL) | 12 ± 1.34 | 15 ± 3.19 | > 0.05 |

Note: Data were analyzed using one-way analysis of variance and a two-sided Student’s *t*-test. Data were presented as the mean ± SD. Testicular volumes were evaluated using ultrasound examination A: Globozoospermia; B: Obstructive azoospermia.
